# INTERGENERATIONAL CREATIVE MOVEMENT INTERVENTION FOSTERS SOCIAL CONNECTIONS AND MENTAL HEALTH

**DOI:** 10.1093/geroni/igae098.0469

**Published:** 2024-12-31

**Authors:** Amy Kostelic, Anthony Bardo, Susie Thiel, Claire Reardon, Mohammad Khalilian

**Affiliations:** University of Kentucky, Lexington, Kentucky, United States; University of Kentucky, Lexington, Kentucky, United States; University of Kentucky, Lexington, Kentucky, United States; University of Kentucky, Lexington, Kentucky, United States; University of Kentucky, Lexington, Kentucky, United States

## Abstract

It is widely accepted that people flourish when experiencing a sense of connection and belonging. Yet, negative outcomes from the pandemic, like loneliness, remain elevated and relevant for younger and older women’s well-being. Additionally, life course transitions into both young adulthood and older age, while very different, are also similar in that they can challenge a woman’s social connectedness and sense of belonging. Our innovative multi-lesson education program aimed to connect first-generation female college students and college educated older women living in the community to promote a sense of belonging and enhance mental health by (a) combining interpretive dance in the form of physical activity and creativity; (b) engaging multiple generations via a humanistic approach centered around generativity; and (c) utilizing a sociocentric network approach to identify individual and program-level factors associated with positive outcomes. Results based on Social Network Analyses (SNA) demonstrated that our program was adept at fostering close intergenerational connections, which when triangulated with qualitative results from in-depth interviews, were found to be anchored in an emergent sense of belonging. Coupled with results from pre- and post-intervention surveys, the formation of these close intergenerational connections was found to positively impact mental health. The efficacy of our program was further established when pre- and post-intervention mental health assessments were compared between the participant (n=20) and control group (n=20). Our innovative program demonstrates the potential, though distinct, applicability of (a) creative movement activities and (b) SNA techniques for developing a programmatic scale up into other community settings.

